# Cacalol Acetate as Anticancer Agent: Antiproliferative, Pro-Apoptotic, Cytostatic, and Anti-Migratory Effects

**DOI:** 10.3390/cimb46090550

**Published:** 2024-08-23

**Authors:** Gareth Omar Rostro-Alonso, Alejandro Israel Castillo-Montoya, Juan Carlos García-Acosta, Erick Fernando Aguilar-Llanos, Laura Itzel Quintas-Granados, Edgar Yebrán Villegas-Vazquez, Rosario García-Aguilar, Samantha Andrea Porras-Vázquez, Lilia Patricia Bustamante-Montes, Jesús J. Alvarado-Sansininea, Manuel Jiménez-Estrada, Lizbeth Cariño-Calvo, Manuel González-del Carmen, Hernán Cortés, Gerardo Leyva-Gómez, Gabriela Figueroa-González, Octavio Daniel Reyes-Hernández

**Affiliations:** 1 Laboratorio de Farmacogenética, Unidad Multidisciplinaria de Investigación Experimental Zaragoza, Facultad de Estudios Superiores Zaragoza, Universidad Nacional Autónoma de México, Batalla 5 de Mayo s/n Esquina Fuerte de Loreto, Iztapalapa, Mexico City 09230, Mexico; rostroalonsogarethomar@gmail.com (G.O.R.-A.); alex24castillomontoy@gmail.com (A.I.C.-M.); jcarlosga99@gmail.com (J.C.G.-A.); eyebran.villegas@gmail.com (E.Y.V.-V.); andy.porras13@gmail.com (S.A.P.-V.); gabriela.figueroa@zaragoza.unam.mx (G.F.-G.); 2 Laboratorio de Biología Molecular del Cáncer, Unidad Multidisciplinaria de Investigación Experimental Zaragoza, Facultad de Estudios Superiores-Zaragoza, Universidad Nacional Autónoma de México, Iztapalapa, Mexico City 09230, Mexico; fernandoaguilarllanos@gmail.com; 3 Colegio de Ciencias y Humanidades, Plantel Cuautepec, Universidad Autónoma de la Ciudad de México, Calle Dr. García Diego 168, Doctores, Cuauhtémoc, Mexico City 06720, Mexico; itzel.quintas@uacm.edu.mx; 4 Laboratorio de Citometría de Flujo y Hematología, Diagnóstico Molecular de Leucemias y Terapia Celular (DILETEC), Gustavo A. Madero, Mexico City 06350, Mexico; rgarcia@diletec.com.mx; 5 Coordinacion de Investigacion, Centro Universitario Siglo XXI, Edo. de Mex, Mexico City 03100, Mexico; liliapatricia.bustamante@cus21.edu.mx; 6 Laboratorio 2–10, Departamento de Productos Naturales, Instituto de Química, Universidad Nacional Autónoma de México, Mexico City 04510, Mexico; javier33@comunidad.unam.mx (J.J.A.-S.); manueljemex@gmail.com (M.J.-E.); 7 Facultad de Ciencias Químicas, Universidad Veracruzana, Orizaba 94340, Mexico; lcarino@uv.mx; 8 Facultad de Medicina, Universidad Veracruzana, Ciudad Mendoza 94740, Mexico; manugonzalez@uv.mx; 9 Laboratorio de Medicina Genómica, Departamento de Genómica, Instituto Nacional de Rehabilitación Luis Guillermo Ibarra Ibarra, Mexico City 14389, Mexico; hcortes@inr.gob.mx; 10 Departamento de Farmacia, Facultad de Química, Universidad Nacional Autónoma de México, Mexico City 04510, Mexico; leyva@quimica.unam.mx

**Keywords:** cacalol, cacalol acetate, antiproliferation, apoptotic effect, cervical cancer cells

## Abstract

Cacalol (C), a sesquiterpene isolated from *Psacalium decompositum*, has demonstrated anti-inflammatory and antioxidant activities. Its cytotoxic, antiproliferative, and pro-apoptotic effects have been previously shown in an in vitro breast cancer model. A derivative, cacalol acetate (CA), shows potential in regulating these processes, which has not been previously reported. This study focused on an in vitro cervical cancer model, assessing CA’s antiproliferative, pro-apoptotic, cytostatic, and anti-migratory activities using the HeLa cell line. The natural anticancer agent indole-3-carbinol (I3C) was used as a control for comparison. CA demonstrated significant antitumor activities, including inhibiting cell growth, inducing apoptosis, arresting cells in the G2 phase of the cell cycle, and inhibiting cell migration. These effects were notably greater compared to I3C. I3C, while following a similar trend, did not induce Cas-3 expression, suggesting a different apoptotic pathway. Neither CA nor I3C increased p62 and LC3B levels, indicating they do not stimulate autophagy marker expression. Both compounds inhibited HeLa cell migration and induced cell cycle arrest. Despite both holding promise as anticancer agents for cervical cancer, CA’s lower cytotoxicity and stronger regulation of tumor phenotypes make it a more promising agent compared to I3C.

## 1. Introduction

According to the World Health Organization (WHO), cervical cancer (CC) is the fourth most frequently occurring cancer worldwide [1] and is one of the leading causes of cancer death among women [2]. CC is the leading cause of cancer-related deaths among women in Eastern, Western, Middle, and Southern Africa. China and India together account for over one-third of the global cervical cancer burden, with 106,000 cases and 48,000 deaths in China and 97,000 cases and 60,000 deaths in India. Globally, the average age at diagnosis for CC is 53 years, making it one of the top three cancers affecting women under 45 years old [1].

CC remains a significant health burden among young women globally, especially in low- and middle-income countries. Between 2014 and 2017, Paraguay and Venezuela reported the highest cervical cancer mortality rates, while Puerto Rico had the lowest. Overall, most Latin American and Caribbean countries, such as Chile, Colombia, Cuba, El Salvador, Mexico, Nicaragua, Panama, and Peru, showed a decline in cervical cancer mortality during this period. Conversely, Brazil and Paraguay exhibited significant increases [3].

Although CC is a preventable disease, many women present with invasive disease requiring radical surgery (early-stage disease) and/or combined radiation therapy and chemotherapy (locally advanced disease), radiosensitizing nanoparticles [4,5], and immunotherapies [6]; nevertheless, these strategies are not successful interventions. To achieve CC elimination goals, there is a need for new programmatic approaches to introduce emerging technologies and scale up the screening and treatment of cervical precancer. Implementation research, which is underutilized, plays a crucial role in facilitating the widespread adoption of evidence-based interventions [7]. Fortunately, a large amount of information dealing with the clinical aspects of cancer chemotherapy has been generated, including the finding and application of natural-origin drugs.

Against this background, there are two principal requirements of research on antitumor chemotherapy that achieves an efficient antitumoral agent and satisfies a broad population spectrum: to find a more efficient natural antitumoral agent without adverse effects and a more profitable source of this kind of agent. In this way, we previously reported that indole-3-carbinol (I3C), a non-carcinogenic agonist compound of the aryl hydrocarbon receptor (AhR), promotes the activation of AhR and decreases cell proliferation, possibly through UBE2L3 (also known as UBCH7, UbcH7, and E2 Ubiquitin-Conjugating Enzyme L3) mRNA induction, which would result in the ubiquitination of HPV (Human Papilloma Virus) E7 protein [8]. However, it is still unknown whether I3C affects other hallmarks of cancer.

**Scheme 1:**
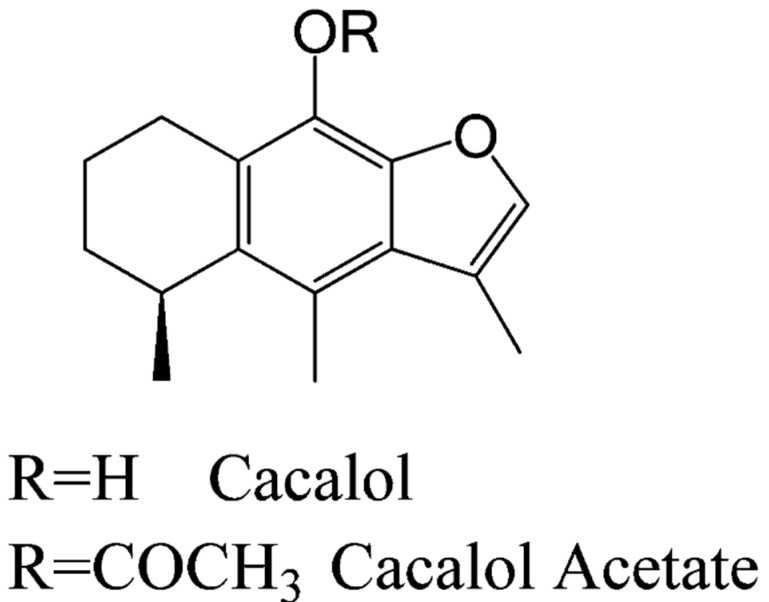
Structure of C and CA. Adapted from Gómez-Vidales and colleagues [14].

On the other hand, another bioactive molecule is cacalol (C), a phytochemical compound with anti-inflammatory and antioxidant properties [9,10,11]. It has a strong antiproliferative effect against breast cancer cells, inducing apoptosis by activating a pro-apoptotic pathway. C inhibits tumor growth in vivo by blocking fatty acid synthase (FAS) gene expression through the modulation of the Akt-SREBP pathway [12]. Cacalol exhibits potential anti-FAS activity and induces apoptosis in breast cancer cells, with possible synergistic effects when combined with cyclophosphamide [13]. Furthermore, its derivative compound, cacalol acetate (CA) (see Scheme 1), has shown anti-inflammatory properties [9] and both C and CA demonstrate potential as photoproducers of singlet oxygen and free radical scavengers, making them promising candidates for new therapeutic applications in the treatment of tumors and other diseases [14]. However, their antitumoral activity is not well known yet.

Although there is no evidence of the antitumoral properties of cacalol acetate in CC, this compound has shown potential utility as a chemopreventive and chemotherapeutic agent against cancer biological models. Particularly, in this study, we investigated the antiproliferative, pro-apoptotic, and anti-migratory properties of CA and I3C in a cervical cancer cell line.

## 2. Materials and Methods

### 2.1. Cell Cultures

The cervical cancer cell line HeLa (positive to HPV-18) was obtained from the American Type Culture Collection (ATCC, Rockville, MD, USA). For all assays, HeLa cells were cultured in RPMI-1640 media (GIBCO, Carlsbad, CA, USA) and supplemented with 5% newborn calf serum (NCS, GIBCO, USA), L-glutamine, red phenol, and benzylpenicillin. Cultures were maintained in an incubator (Nauire, Plymouth, MN, USA) with a humidified atmosphere at 5% CO_2_ and 37 °C. All cell-based assays were performed using culture cells in the exponential growth phase.

### 2.2. Tested Compounds

CA was solubilized in 100 µL dimethyl sulfoxide (DMSO; Sigma-Aldrich, St. Louis, MO, USA) and 900 µL of RPMI-1640 medium, resulting in a final concentration of 1 µg/µL. I3C (Sigma-Aldrich, St. Louis, MO, USA; purity ≥96%) was solubilized in DMSO and used at a final concentration of 150 µM.

### 2.3. Cell Proliferation Assays

HeLa cells (6 × 10^3^ cell/well) were cultivated in a 96-well tissue culture plate (Corning, New York, NY, USA) with 100 µL of RPMI-1640 medium and growth for 24 h at 37 °C and 5% CO_2_. Then, CA was added at several concentrations (from 20 µg/mL to 29 µg/mL) and incubated as mentioned above. I3C was added at a final concentration of 150 µM. As controls, we added 5 µg/mL of DMSO (vehicle) in a cell culture sample and included one cell culture without treatment (control). In addition, HeLa cells were treated with 1 µM of beta-naphthoflavone (BNF), an agonist synthetic ligand of AhR with anticancer activity against mammary carcinoma cells [15] and cervical cancer cells [16] as a control. After 24 h of incubation with CA, we determined the antiproliferative activity (IC_50_) by crystal violet staining as previously reported [17,18]. Finally, the cell count was performed spectrophotometrically at 590 nm (Awareness Technology INC, Chromate 4300, Palm City, FL, USA). Data were analyzed in a dose–response curve to estimate the concentration at which 50% of the cell population decreases (IC_50_).

### 2.4. Determination of Apoptosis by Evaluation of Active Caspase-3

HeLa cells (6 × 10^3^ cell/well) were incubated for 24 h with 102.72 μM of CA and I3C (150 µM). As controls, cells were incubated with 5 µg/mL DMSO (vehicle), 1 mg/mL colchicine (positive apoptotic cell death control), and beta-naphthoflavone (BNF) (1 µM). For the immunodetection of active caspase-3, we followed a reported protocol [18]. Briefly, cells were permeabilized with Triton X-100 (1%) for 20 min, after which the cells were washed with phosphate-buffered saline (PBS) pH 7.3. Then, the cells were incubated with rabbit anti-human active caspase-3 polyclonal antibody (Sigma-Aldrich, St. Louis, MO, USA) diluted in PBS (1:1000) for 18 h at 4 °C. Following this, the cells were washed and incubated with goat anti-rabbit IgG secondary antibody coupled to fluorescein-5-isothiocyanate (FITC) diluted 1:1000 in PBS at room temperature for 2 h. Then, the samples were stained with 4′6-diamidino-2-phenylindole (DAPI) and analyzed under epifluorescence microscopy (Eclipse E600, Nikon, Melville, NY, USA) and phase-contrast microscopy (Eclipse TS2R-FL, Nikon, Japan). We used a DXMI200F digital camera (Nikon, Melville, NY, USA) for recorded images.

### 2.5. Determination of Autophagic (p62 and LC3B) Biomarkers by Western Blot Analysis

HeLa cells treated with CA (102.72 μM), I3C (150 µM), DMSO (5 µg/mL, vehicle), and BNF (1 µM) and untreated cells (control) were grown for 24 h and were subjected to lysis using RIPA buffer (Santa Cruz Biotechnology, Inc., Dallas, TX, USA, sc-24948) supplemented with 0.1% protease inhibitors (Complete Protease Inhibitor Cocktail, Roche, Kansas City, MO, USA, catalog number 11697498008). The lysates were then incubated at 4 °C for 30 min, followed by centrifugation at 13,000× *g* at 4 °C for 25 min. The soluble protein concentrations in the cell lysates were measured spectrophotometrically at 280 nm using the EPOCH Microplate Spectrophotometer (Bio Tek, Winooski, VT, USA). Equal amounts of protein from each sample were combined with 5X Laemmli sample buffer (10% SDS, 50% glycerol, 0.02% bromophenol blue, and 0.3125 M Tris HCl, pH 6.8), supplemented with β-mercaptoethanol, and boiled for 10 min. Subsequently, 50 µg of protein from each sample was separated on 12% SDS-PAGE gels using a vertical electrophoresis system (Mini Trans-Blot^®^ Cell, Bio-Rad, Hercules, CA, USA, catalog number 1703810). The separated proteins were then transferred onto 0.45 µm polyvinylidene difluoride (PVDF) membranes (Thermo Scientific, Waltham, MA, USA, catalog number 88518) using a Trans-Blot Turbo chamber (Bio-Rad) at 25 V and 1 mA for 30 min. The membranes were subsequently blocked with 5% non-fat milk in TBS (pH 7.0) containing 1% Tween-20 for 2 h at room temperature, followed by incubation with primary antibodies against p62 Rabbit pAb (1:3000 dilution, ABclonal, Woburn, MA, USA), LC3B Rabbit pAb (1:3000 dilution, ABclonal), and β-actin mouse mAb (1:10,000 dilution, ABclonal, catalog number AC004) overnight at 4 °C on a rocking platform. After washing the membranes five times with TBS (pH 7.0) containing 0.1% Tween-20, the membranes were incubated for 1 h at 25 °C with peroxidase-conjugated secondary antibodies anti-rabbit IgG (1:10,000 dilution, Cell Signaling Biotechnology, Danvers, MA, USA, catalog number 7074s) or anti-mouse IgG (1:10,000 dilution, Cell Signaling Biotechnology, catalog number 7076s) for β-actin detection. Finally, the membranes were washed with TBS (pH 7.0) containing 0.1% Tween-20 and developed using chemiluminescence with Clarity MaxTM Western ECL (Bio-Rad, catalog number 1705062) following the manufacturer’s instructions. Image analysis was performed using the C-DiGit Blot (LI-COR, Lincoln, NE, USA). All experiments were conducted in triplicate. β-actin was consistently detected as a loading control and used for normalizing the densitometry of the target proteins with ImageJ bundled with Java 8 software. Data normalization was carried out by dividing the densitometry data of the target protein by that of the loading control protein (β-actin).

### 2.6. Determination of Cell Migration

To assess cell migration, a wound healing assay was conducted under sterile conditions. In 96-well plates, adhesive tape (0.5 mm width) was carefully positioned, and a mark with a 2-mm division was applied at the center of each well to demarcate the area for subsequent photographic analysis. Following this, HeLa cells were seeded at a density of 3 × 10^4^ cells per well in 100 µL of RPMI medium supplemented with 10% neonatal serum and incubated for 24 h at 37 °C with 5% CO_2_. After the initial incubation period, 10 µM cytarabine (ara-C) was added and cells were further incubated for 2 h under the same conditions. The adhesive tape was then carefully removed to create a wound. The samples were gently washed with PBS (pH 7.0), and HeLa cells were treated with CA (102.72 μM), I3C (150 µM), DMSO (5 µg/mL, vehicle), and BNF (1 µM). Negative controls (untreated cells) and cells treated with 105 mM TGF-β were included in each experimental set. Cell migration was observed at various time points (0, 24, 48, and 72 h) and documented using a Canon camera. Each experiment was conducted in triplicate, with three biological replicates and technical triplicates for each measurement. The thickness of the wound was analyzed using bioinformatics tools (ImageJ bundled with Java 8) by assessing images captured at different time points within the designated area and quantifying the migration rate of the cells.

### 2.7. Determination of Cell Cycle Arrest

Cells (3 × 10^5^) were seeded in a 6-well plate and incubated for 24 h at 37 °C with 5% CO₂. Subsequently, the cells were divided into untreated and treated groups. Treatment involved the addition of 20 µL of colchicine (1 mg/mL, as a positive control), 102.72 μM of CA, 150 µM of I3C, 5 µg/mL DMSO (vehicle), and 1 µM of BNF. The cells were then incubated for an additional 24 h at 37 °C with 5% CO_2_. Following the incubation period, the cells were trypsinized and collected by centrifugation at 212× *g* for 3 min at room temperature. The collected cells were fixed in ice-cold methanol (500 µL) with PBS (pH 7.0) (500 µL) at 4 °C for 1 h. After fixation, the cells were washed three times with PBS (pH 7.0) (1 mL each wash) and centrifuged as described above. Next, the cells were treated with 30 µL of RNase A (100 U/mL) and incubated for 30 min at 37 °C. After another round of centrifugation, the cells were resuspended in 500 µL of PBS containing 5 µL of propidium iodide (20 µg/mL) and analyzed using the Beckman-Coulter CytoFlex cytometer (Beckman Coulter, Brea, CA, USA). The concentration of colchicine chosen for treatment was based on reported values known to arrest the cell cycle at the G2 phase. Each measurement was conducted with three biological replicates and technical duplicates.

### 2.8. Statistical Analysis

All data were reported in terms of means and standard errors (SEs). Statistical analyses were performed by variance differences (ANOVA), followed by Holm–Sidak test and Student’s *t*-test, using GraphPad Prism 9.0.2.

## 3. Results

### 3.1. Cytotoxicity Activity of Phytochemical Compounds on HeLa Cells

**Figure 1:**
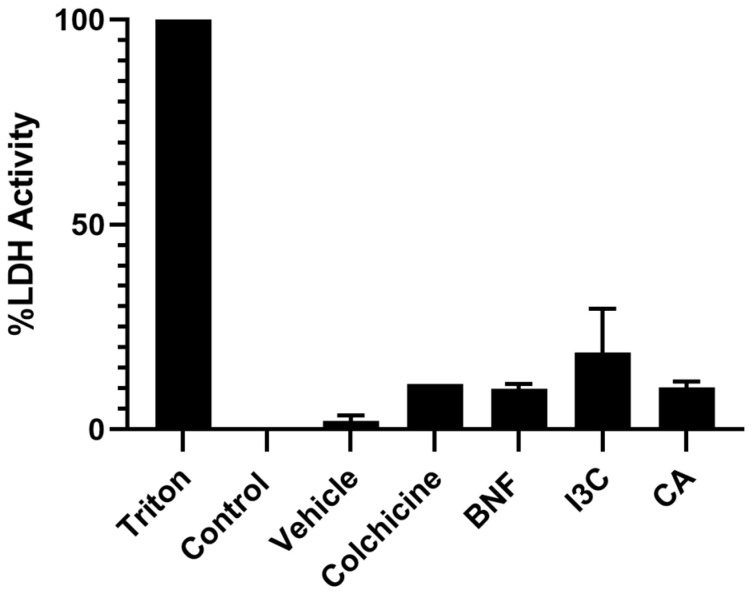
Cytotoxicity effect of CA and I3C on HeLa cells. HeLa cells treated with CA (102.72 μM), I3C (150 µM), DMSO (5 µg/mL, vehicle), BNF (1 µM), and Triton X-100 (positive control) and untreated cells (control) were analyzed by LDH assay. Bars indicate the percentage of LDH activity in the presence of tested compounds. Experimental data show the means ± standard errors (SEs) of three independent assays performed in triplicate. Statistically significant differences were calculated according to ANOVA test followed by Tukey’s test (*p* < 0.05 vs. control).

First, we determined cellular cytotoxicity by measuring lactate dehydrogenase (LDH), an enzyme that catalyzes the conversion of lactate to pyruvate and is released from cells after membrane dissolution by a toxic stimulus. We did not find cytotoxic activity on HeLa cells from CA or I3C at concentrations of 102.72 μM or 150 μM, respectively, compared to the untreated control or vehicle (Figure 1).

### 3.2. Antiproliferative Activity of CA and I3C in HeLa Cells

**Figure 2:**
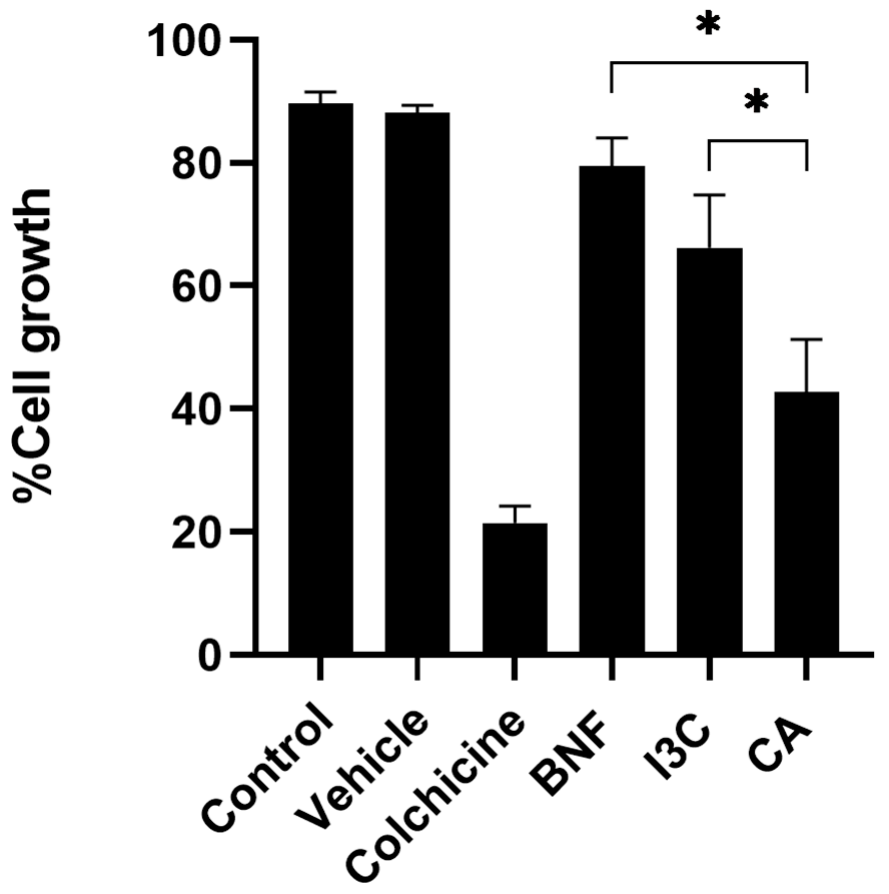
Effect of CA and I3C on cell growth. HeLa cells treated with CA (102.72 μM), I3C (150 µM), DMSO (5 µg/mL, vehicle), BNF (1 µM), or colchicine (25.03 µM) and untreated cells (control) were analyzed by crystal violet staining. Bars indicate the cell growth percentage in the presence of tested compounds. Experimental data show the means ± standard error (SE) of three independent antiproliferative assays performed in triplicate. All groups showed a statistically significant difference when compared with the control and vehicle groups (*p* < 0.001, Holm–Sidak test), as well as when compared with colchicine (*p* < 0.002, Holm–Sidak test). We also observed a statistically significant difference when comparing CA with BNF or I3C (* *p* < 0.001, Holm–Sidak test).

The antiproliferative effect of CA and I3C on HeLa cells was determined. Antiproliferative activity was measured by a decrease in cell growth 24 h after compound administration. Both compounds had the ability to impair HeLa cell proliferation (Figure 2). A concentration of 150 µM of I3C resulted in 66% ± 8.6% of cell growth. In contrast, CA (102.72 μM) resulted in 43% ± 8.5% of cell growth. Taken together, these results suggest that CA has greater antiproliferative activity for HeLa cells than I3C.

### 3.3. Caspase-3-Induced-Apoptosis in HeLa Cells by CA and I3C

**Figure 3:**
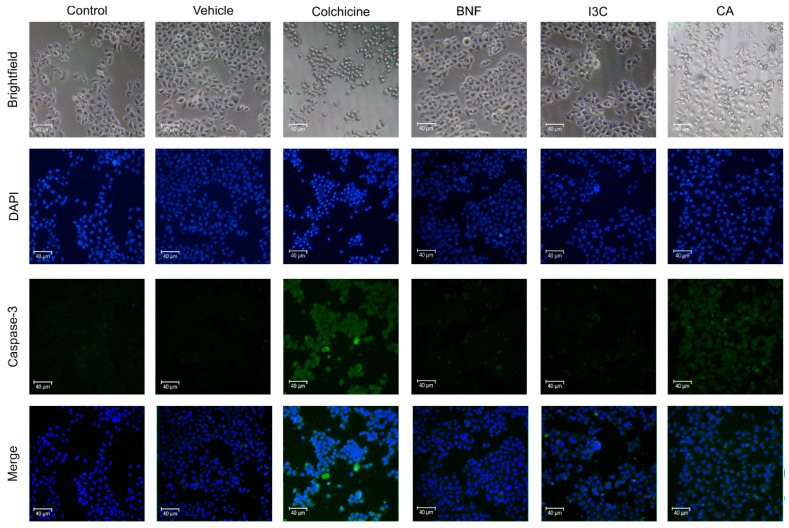
Confocal microscopy of pro-apoptotic Cas-3 protein in HeLa cells. HeLa cells treated with CA (102.72 μM), I3C (150 µM), DMSO (5 µg/mL, vehicle), BNF (1 µM), and colchicine (25.03 µM) or untreated cells (control) were used for immunodetection of caspase-3 (green fluorescence) with DAPI-counterstained nuclei (blue fluorescence).

The apoptotic pathway and its initiation may rely on the liberation of cytochrome c and caspase-9 activation, resulting in caspase-3 cleavage [19]. To determine the correlation between Cas-3-induced-apoptosis by CA or I3C, we treated HeLa cells with those compounds (Figure 3). We observed that CA induces the expression of Cas-3, suggesting that CA induces apoptosis via Cas-3. Moreover, after I3C and BNF treatments, the expression of Cas-3 was not increased, suggesting that Cas-3-induced-apoptosis was not triggered by I3C or BNF. The positive control (colchicine) induces Cas-3 expression, thereby activating the Cas-3-induced-apoptosis.

### 3.4. Determination of Autophagy Induced by CA and I3C Treatments in HeLa Cells

**Figure 4:**
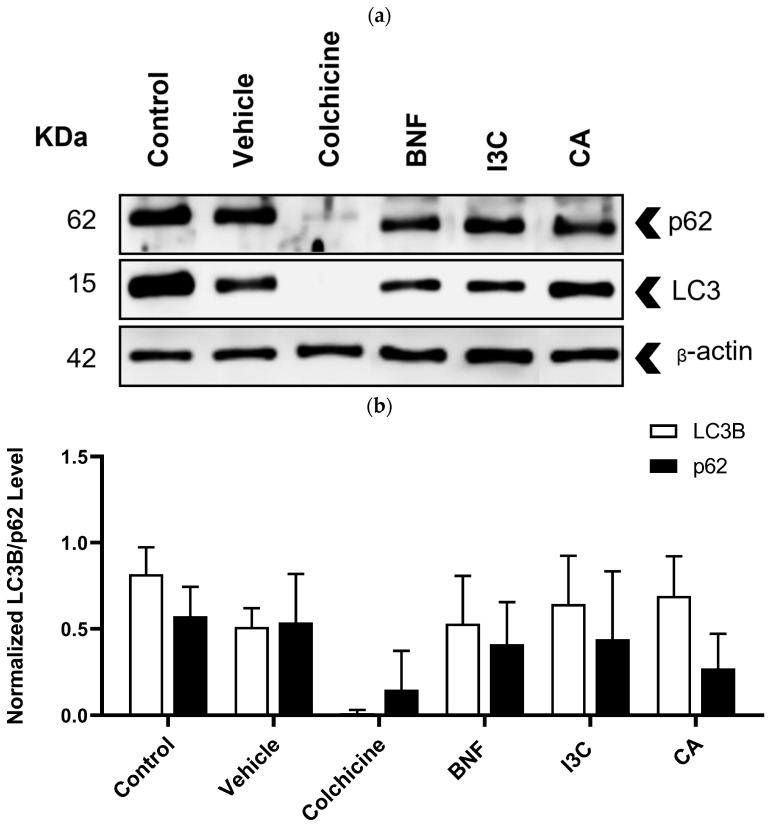
Western blot analysis to determine LC3B and p62 proteins in HeLa cells. (**a**) HeLa cells treated with CA (102.72 μM), I3C (150 µM), DMSO (5 µg/mL, vehicle), BNF (1 µM), colchicine (25.03 µM) and untreated cells (control) were used to detect p62 (62 kDa), LC3B (15 kDa), and β-actin (42 kDa). (**b**) Protein expression levels of p62 and LC3B were normalized using β-actin expression (loading control). Bars indicate the mean levels of protein expression ± standard error (SE) of three independent antiproliferative assays performed in triplicate.

Autophagy biomarkers such as LC3B and p62 were detected by immunoblotting (Figure 4a) and the expression levels of LC3B and p62 were normalized using the expression of β-actin as a loading control (Figure 4b). In all treatments, the expression levels of LC3B or p62 were not statistically significantly different. Neither CA, I3C, nor BFN induced changes in the expression of LC3B or p62, suggesting that these compounds did not induce autophagy.

### 3.5. Effect of CA and I3C on Cell Cycle Arrest of HeLa Cells

**Figure 5:**
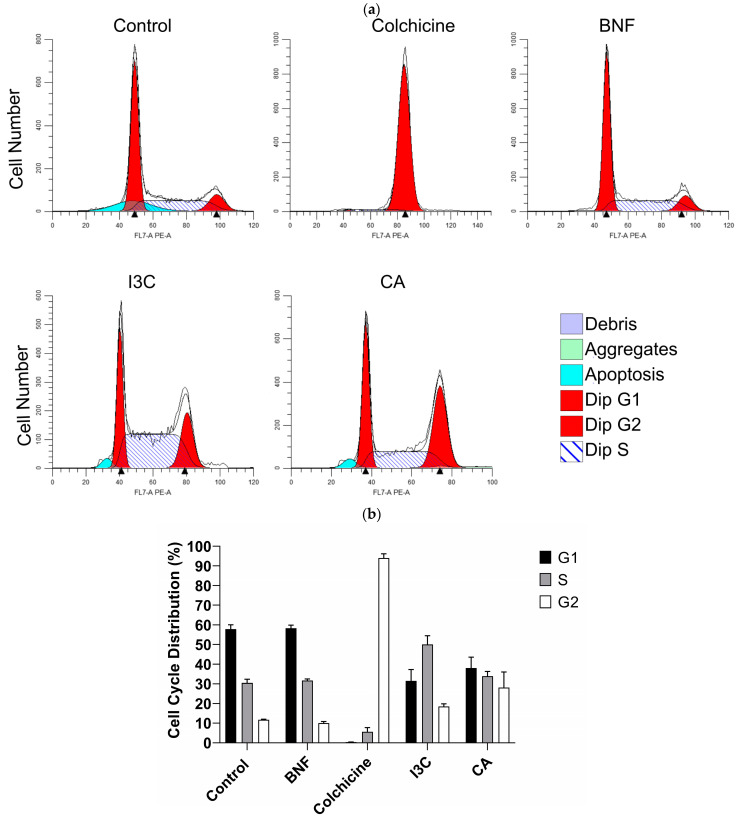
Effect of CA and I3C on cell cycle arrest of HeLa cells. (**a**) Flow cytometry graphs depicting the cell cycle distribution of HeLa cells treated with CA (102.72 μM), I3C (150 µM), BNF (1 µM), and colchicine (25.03 µM). (**b**) The percentages of cells in the G1, S, and G2 phases of the cell cycle for treated HeLa cells.

To explore the impact of CA and I3C on cell cycle progression, we examined the distribution of HeLa cells treated with both compounds (Figure 5a). Approximately 28% of HeLa cells treated with CA remained in the G2 phase. In contrast, 18% of cells treated with I3C remained in this phase. Therefore, CA has a stronger effect on arresting the cell cycle of HeLa cells in phase 2 than I3C. Additionally, HeLa cells treated with I3C predominantly remained in the S phase (48%), while 58% of cells treated with BNF remained in the G1 phase. Approximately 95% of cells treated with colchicine (positive control) remained in the G2 phase (Figure 5b).

### 3.6. Effect of CA and I3C on Cell Migration of HeLa Cells

**Figure 6:**
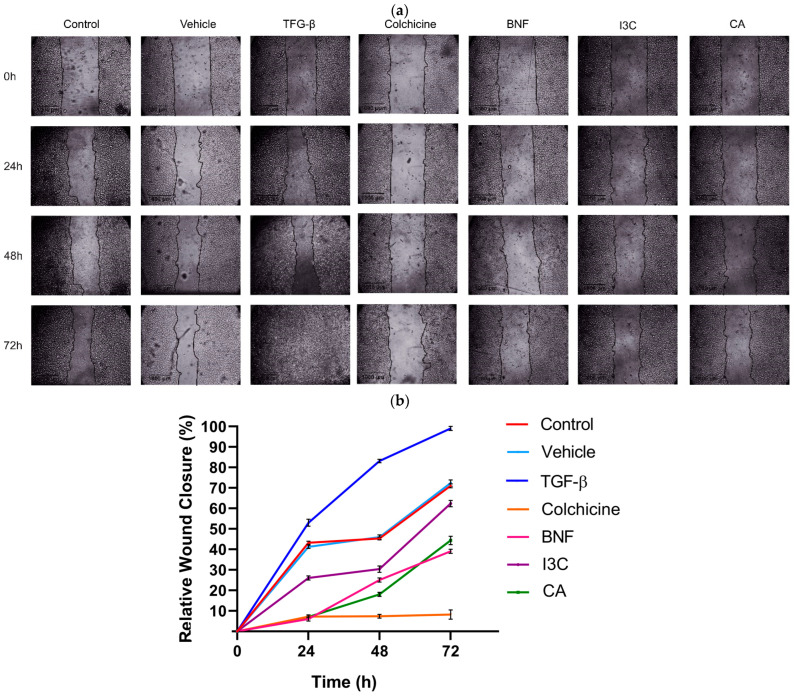
Effect of CA and I3C on cell migration of HeLa cells. (**a**) HeLa cells were subjected to treatment with CA (102.72 μM), I3C (150 µM), DMSO (5 µg/mL, vehicle), BNF (1 µM), TGF-β (105 mM), and colchicine (25.03 µM) and compared to untreated cells (control). Microscopic images were captured at various time intervals, including 0 h, 24 h, 48 h, and 72 h, to visualize the wound closure. The migrating cell edges were delineated by lines. (**b**) Relative wound closure percentage was calculated for all experimental conditions. The lines represent the mean of three independent measurements, with the standard deviation indicated on each bar. Statistical analysis was conducted to compare the experimental conditions with the negative control (*p* < 0.05).

Wound assays were conducted to assess whether CA or I3C could influence cell migration in HeLa cells (Figure 6a,b). At 72 h, the cell migration of HeLa cells was significantly enhanced following TGF-β treatment (positive control) compared to untreated cells [negative control, C(-)] and vehicle. Conversely, the presence of CA significantly reduced cell migration by 58% in HeLa cells at 72 h (*p* > 0.05) compared to the positive control. On the other hand, in HeLa cells, relative migration decreased by 40% after 72 h of treatment with 150 µM I3C compared to the positive control. Additionally, BNF reduced HeLa cell migration by 75%, while the negative control and vehicle reduced cell migration by approximately 30% compared to the positive control. These results suggested that CA (102.72 μM) reduced HeLa cell migration 1.45-fold more than I3C did. Therefore, CA has a greater effect on reducing the cell migration of HeLa cells compared to I3C.

## 4. Discussion

Sesquiterpenes present in several plant species induce apoptosis in cancer cells through different mechanisms [20]. It has been reported that C has shown anticancer properties inducing apoptosis in several cancer cells, particularly in breast cancer cells.

Our data showed that CA had no cytotoxic effects on HeLa cells and that this compound exhibited antiproliferative activity in a dose-dependent manner. The IC_50_ value of cacalol acetate for HeLa cells was 102.72 μM, whereas for I3C, it was 150 μM, suggesting the strong potential of CA as an anticancer agent through cell growth inhibition. In breast cancer cells (MCF7 and MDA-MD231) and in a xenograft mouse model, cacalol inhibits cell growth without toxic effects and significantly suppresses tumor growth when administered intraperitoneally or orally, suggesting its potential as a preventive and therapeutic agent against cancer [12]. Cacalol and its derivative, CA, have antioxidant activity due to their ability to act as potent free radical scavengers, contributing to their anticancer properties by reducing oxidative stress in cancer cells [14]. Moreover, this phytochemical compound inhibits the lipid peroxidation induced by free radicals [10].

HeLa cells treated with CA showed increased levels in the expression of caspase-3. In contrast, when cells were treated with I3C, the expression of this apoptotic biomarker was not observed, suggesting that CA induces apoptotic cell death via Cas-3, whereas I3C does not. These results are consistent with our previous report, in which we measured apoptosis in I3C-treated HeLa cells by cytometry and did not observe increased levels of apoptosis [8]. Our evidence suggested that the induction of apoptosis by CA is independent of Ahr. However, the molecular mechanism of CA is still unknown. Previous reports suggest that cacalol has an anti-breast cancer effect by inhibiting fatty acid synthase (FAS) at transcriptional and post-transcriptional levels and modulating the Akt-SREBP (sterol regulatory element-binding protein) pathways. In this way, cacalol blocks P13K/Akt signaling resulting in an inhibition of SREBP1, which is the main transcriptional regulator of FAS [12]. Therefore, cacalol induces apoptosis in breast cancer cells by modulating the Akt-SREBP-FAS signaling pathway, leading to the activation of pro-apoptotic proteins DAPK2 and caspase 3 [12]. Furthermore, cacalol inhibits the FAS gene, essential in fatty acid biosynthesis and energy homeostasis, causing apoptosis through its antioxidant activity [13]. Intrinsic apoptosis is a cell death centered in the mitochondrion [21]. The activation of Bax and Bak (BCL-2 family members) results in mitochondrial outer membrane permeabilization (MOMP) and the releasing of pro-apoptotic proteins and cytochrome *c* from the inter-membrane mitochondrial space into the cytosol, where cytochrome *c* binds to Apaf-1, forming an apoptosome and activating caspase-9, which cleaves and activates caspase-3 and -7 [22,23,24,25,26]. Therefore, CA induces apoptotic cell death through promoting more cervical cancer cells expressing caspase-3. The biomarker of late stage of apoptosis (caspase-3) was not significantly upregulated in HeLa cells treated with CA treatment. Previous reports indicated that the treatment of tumor cells with cacalol promote the expression of DAPK2 and caspase-3 [12]. Our results agree, showing that CA induces the expression of caspase-3, suggesting that the acetylation of cacalol might enhance the apoptotic induction mechanism of this compound. Although some evidence suggests that cacalol induces apoptosis through the mechanisms described above, the acetylated molecule (CA) might trigger apoptosis through a different pathway, which we have elucidated.

Furthermore, cacalol has synergistic activity that enhances the apoptotic effect of chemotherapeutic drugs such as taxol and cyclophosphamide, helping to overcome chemoresistance [12]. It is still unclear if CA might have synergistic activity.

**Figure 7:**
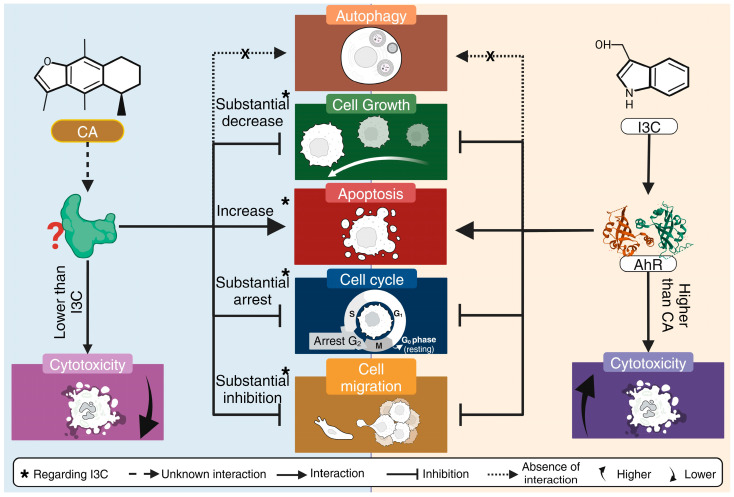
Summary of the anticancer activity of CA and I3C on HeLa cells. CA induces a substantial decrease in cell growth in comparison to I3C and substantially inhibits cell migration in comparison to I3C. CA induces apoptosis and arrests the cell cycle at a higher rate than I3C. CA and I3C do not induce autophagy.

In addition, neither CA nor I3C induce autophagic cell death. CA arrests the cell cycle at G2 phase, while I3C also arrests the cell cycle at this phase, albeit to a lesser extent. CA could inhibit cell migration and has a relatively low cytotoxicity, as it does not affect HeLa cell morphology as does BNF (1uM). It is worth mentioning that CA does not cause a morphological cytotoxic effect associated with its ability to inhibit cell migration (Figure 7).

## 5. Conclusions

CA had a lower cytotoxic effect on HeLa cells than I3C. CA and I3C inhibited cell growth, but CA is more effective in inhibiting cell proliferation than I3C. I3C did not affect the expression of Cas-3, suggesting that this compound did not induce apoptosis via Cas-3. In contrast, CA upregulated the expression of Cas-3, suggesting that CA induces apoptosis via Cas-3. CA and I3C did not induce the expression of p62 and LC3B levels, suggesting that these phytochemical compounds not induce autophagic cell death. CA arrests the cell cycle and inhibits the cell migration of HeLa cells at a higher rate than I3C. Although CA and I3C are promising anticancer agents for the treatment of cervical cancer, the low cytotoxicity of CA compared to I3C and the fact that CA induces apoptosis via Cas-3 and cell cycle arrest and inhibits cell proliferation and migration at a higher rate than I3C make CA a more promising agent than I3C.

## Data Availability

The original contributions presented in this study are included in the article; further inquiries can be directed to the corresponding author.
